# Clonality, virulence genes, and antibiotic resistance of *Staphylococcus aureus* isolated from blood in Shandong, China

**DOI:** 10.1186/s12866-021-02344-6

**Published:** 2021-10-18

**Authors:** Xuezhi Wang, Dongzi Lin, Zengqi Huang, Jinmei Zhang, Wenyan Xie, Pen Liu, Huaiqi Jing, Jiazheng Wang

**Affiliations:** 1 Department of Laboratory Medicine, Foshan Fourth People’s Hospital, Foshan, 528000 Guangdong China; 2 Department of Clinical Laboratory Medicine, The First Affiliated Hospital of Shandong First Medical University & Shandong Provincial Qianfoshan Hospital, Shandong Medicine and Health Key Laboratory of Laboratory Medicine, Jinan, 250014, Shandong, China; 3 State Key Laboratory for Infectious Disease Prevention and Control, National Institute for Communicable Disease Control and Prevention, Changping, Beijing, 102206 People’s Republic of China

**Keywords:** *Staphylococcus aureus*, Blood, Antibiotic resistance, Virulence genes, Molecular characterization

## Abstract

**Background:**

Bloodstream infection (BSI) caused by *Staphylococcus aureus* (*S. aureus*) can be life-threatening and pose a great challenge to infection control and clinical treatment. However, little information exists regarding the characterization of *S. aureus* in BSI patients in Shandong, China. To identify the clonality, virulence genes, and antibiotic resistance of *S. aureus* in blood, a total of 101 nonrepetitive blood isolates were collected. The antibiotic resistance phenotypes were determined, and virulence genes were analyzed with polymerase chain reaction (PCR). Finally, the genetic relatedness was investigated with Staphylococcus chromosomal cassette *mec* (SCC*mec*) typing for methicillin-resistant *S. aureus* (MRSA) isolates, Staphylococcal protein A (*spa*), and multilocus sequence typing (MLST) for all of 101 isolates.

**Results:**

Of the 101 *S. aureus* isolates, 24 MRSA isolates and 77 methicillin-susceptible *S. aureus* (MSSA) isolates were identified. Overall, MRSA isolates had higher resistance rates than MSSA isolates when exposed to any of the 15 antibiotics tested in this study except for trimethoprim/sulfamethoxazole. Among the 17 virulence genes tested in this study, *hla*, *hld*, and *hlg* could be detected in all isolates. MRSA isolates were more likely to carry *seb* and *hlb* genes, while MSSA isolates were more likely to carry *seg* and *sei* genes. Thirty-five sequence types (STs) and 49 *spa* types were identified, of which ST59-t437 and ST398-t571 were the most abundant. These two genotypes were also the most abundant ST-*spa* types in MRSA and MSSA isolates, but their abundances shifted over time, with ST398-t571 being the predominant genotype from 2016 to 2017, and ST59-t437 from 2018 to 2020. Besides, all the ST59-t437 isolates harbored *hlgb* gene, whereas most (88.9%) ST398-t571 did not. In addition, twenty-four MRSA isolates were subject to SCC*mec* typing. SCC*mec* IVa was the most prevalent SCC*mec* type, and all the ST59-t437 MRSA isolates were SCC*mec* IVa. We also observed 15 new STs, and some of them were MRSA.

**Conclusion:**

These findings provide additional observations and epidemiological data for blood *S. aureus* isolates, which can improve future infection-control measures and aid in potential clinical treatments in hospitals and other clinical settings.

**Supplementary Information:**

The online version contains supplementary material available at 10.1186/s12866-021-02344-6.

## Background

*Staphylococcus aureus* is one of the most infamous and widespread bacteria that cause several diseases, ranging from minor infections of the skin to wound infection, necrotizing pneumonia, and bloodstream infection (BSI) [1, 2]. Among them, BSI can result in high mortality rates and has shown a great impact on health care operations [3, 4]. With the application of invasive diagnostic tests and organ transplantation, the BSI incidence is on the rise [5]. Previous studies have shown that *S. aureus* is the second leading cause of BSI worldwide [6] and the fourth cause of BSI in China [7]. Due to frequently occurring antibiotic resistance in *S. aureus* isolates, especially the occurrence of methicillin-resistant *S. aureus* (MRSA) isolates, *S. aureus* infections are particularly problematic [8]. Compared to methicillin-susceptible *S. aureus* (MSSA), MRSA infections are prone to yield more severe clinical outcomes and are accompanied by an increase in mortality, morbidity, and hospital stay [9, 10], posing great difficulties for clinical treatment.

*S. aureus* produces a variety of virulence factors, including Panton-Valentine leukocidin (PVL), hemolysins (α, β, γ, and δ), toxic shock syndrome toxin-1 (TSST-1), exfoliative toxins (ETs), and staphylococcal enterotoxin (SE) [11]. Among them, hemolysins and PVL are two types of cytolytic toxins which lyse erythrocytes or leukocytes by the cytolytic effects [12]. Both SEs and TSST are staphylococcal superantigens toxins that mainly cause food poison and toxic shock syndrome, respectively [13]. ETs are epidermolytic toxins and are often related to epidermal infections such as bullous impetigo as well as generalized diseases such as Staphylococcal-scalded skin syndrome (SSSS) [14]. Therefore, it is important to reveal the toxin gene profile of *S. aureus* and improve our understanding of its epidemiology.

Epidemiological research of *S. aureus* has indicated that the molecular characteristics of *S. aureus* may change over time and the population structure has regional differences. For example, two sequence types (STs), ST8 and ST121, were the most abundant *S. aureus* in the United States [15], but in the United Kingdom, ST22 was predominant [16] and in France ST398 [17]. In China, ST239-t30 and ST7-t091 were the most prevalent genotypes among MRSA and MSSA isolates, respectively in 2012 [18]. However, from 2013 to 2016, the predominant MRSA genotype was changed from ST239-t30 to ST59-t437 [19]. In 2018, another study found that the most common genotypes among MRSA and MSSA isolates were ST5-t2460 and ST188-t189 respectively [20]. However, scarce data on *S. aureus* BSIs are available from Shandong, China.

In this work, we characterized 101 *S. aureus* strains. Our work aims to elucidate the characterization of virulence genes, evolutionary relationship, and antibiotic resistance of *S. aureus* strains isolated from the bloodstream in a hospital in Shandong province.

## Results

### Antimicrobial susceptibility test

The antimicrobial susceptibility profiles of 101 *S. aureus* strains are shown in Table 1 and Additional file 1. All 101 isolates were susceptible to vancomycin, linezolid, and tigecycline, whereas most of them were resistant to penicillin (85.1%), erythromycin (81.2%), and clindamycin (78.2%). Twenty-four isolates showed cefoxitin and oxacillin (OXA) resistance and all of them harbored *mecA* gene. These isolates were classified as MRSA isolates. Only one MRSA isolate was susceptible to erythromycin, and only two MRSA isolates were susceptible to clindamycin. The statistical analysis showed that MRSA isolates had significantly higher resistance rates than MSSA isolates in penicillin, cefoxitin, oxacillin, tetracycline, and rifampicin (*P* < 0.05). Less than 25% isolates were resistant to the remaining antibiotics.

**Table 1:** Resistance rates of *S. aureus* isolates obtained from blood

Antimicrobial	Resistance rate (%)			*P*-value
	Overall	MRSA	MSSA	
	(*n* = 101)	(*n* = 24)	(*n* = 77)	
Penicillin	86(85.1)	24(100)	62(80.5)	0.044
Cefoxitin	24(23.8)	24(100)	0(0)	0.000
Oxacillin	24(23.8)	24(100)	0(0)	0.000
Gentamicin	15(14.9)	5(20.8)	10(13.0)	0.538
Ciprofloxacin	21(20.8)	8(33.3)	13(16.9)	0.148
Levofloxacin	21(20.8)	8(33.3)	13(16.9)	0.148
Moxifloxacin	17(16.8)	7(29.2)	10(13.0)	0.124
Erythromycin	82(81.2)	23(95.8)	59(76.6)	0.071
Clindamycin	79(78.2)	22(91.7)	57(74.0)	0.068
Linezolid	0(0)	0(0)	0(0)	–
Vancomycin	0(0)	0(0)	0(0)	–
Tetracycline	23(22.8)	14(58.3)	9(11.7)	0.000
Tigecycline	0(0)	0(0)	0(0)	–
Rifampicin	3(3.0)	3(12.5)	0(0)	0.014
Trimethoprim/Sulfamethoxazole	22(21.8)	2(8.3)	20(26.0)	0.068
MDR	77(76.2)	23(95.8)	54(70.1)	0.010

77 (76.2%) isolates were found to be multidrug resistant (MDR) bacteria (i. e. resistant to no less than three classes of antibiotics), of which 23 were MRSA isolates and 54 were MSSA isolates. The statistical analysis showed that the prevalence of MDR was significantly higher in MRSA isolates than in MSSA isolates (*P* < 0.05). Besides, most (89.6%, 69/77) of these isolates showed co-resistance to penicillin, erythromycin, and clindamycin. Among them, the MDR-MRSA isolates had the highest co-resistance rate to five antibiotics “penicillin- cefoxitin-oxacillin-erythromycin-clindamycin” (8/23, 34.8%), whereas the MDR-MSSA isolates had the highest co-resistance rate to “penicillin-erythromycin-clindamycin” (24/54, 44.4%).

### Virulence genes

The *seg* and *sei* genes were the most frequently detected enterotoxin genes, both with a detection rate of 36.6% (37/101, Table 2). In contrast, *see* and *seh* genes were only detected in two MSSA isolates and no MRSA isolates. The *hla*, *hld*, and *hlg* genes could be detected in all 101 isolates, but the detection rate of *hlb* was only 36.6%. The exfoliative toxin genes (*eta* and *etb*) had relatively low detection rates. Only two isolates harbored the *eta* gene and no isolates carried the *etb* gene. Statistically, the prevalence of the toxin genes *seb*, *seg*, *sei*, and *hlb* was significantly different between MRSA and MSSA isolates. The *seb* and *hlb* genes were more likely to be detected in MRSA isolates, and *seg* and *sei* genes were more likely detected in MSSA isolates (Table 2).

**Table 2:** Prevalence of virulence genes of *S. aureus* isolates obtained from blood

gene	Total (*n* = 101) n(%)	distributing in		*P-*value
		MRSA (*n* = 24) n(%)	MSSA (*n* = 77) n(%)	
*eta*	2(2.0)	0(0)	2(2.6)	1.000
*etb*	0(0)	0(0)	0(0)	–
*sea*	14(13.9)	6(25.0)	8(10.4)	0.141
*seb*	33(32.7)	12(50.0)	21(27.3)	0.038
*sec*	13(12.9)	3(12.5)	10(13.0)	1.000
*sed*	33(32.7)	4(16.7)	29(37.7)	0.056
*see*	2(2.0)	0(0)	2(2.6)	1.000
*seg*	37(36.6)	4(16.7)	33(42.9)	0.020
*seh*	2(2.0)	0(0)	2(2.6)	1.000
*sei*	37(36.6)	4(16.7)	33(42.9)	0.020
*sej*	33(32.7)	5(20.8)	28(36.4)	0.157
*hla*	101(100)	24(100)	77(100)	–
*hlb*	37(36.6)	17(70.8)	20(26.0)	0.000
*hld*	101(100)	24(100)	77(100)	–
*hlg*	101(100)	24(100)	77(100)	–
*pvl*	22(21.8)	5(20.8)	17(22.1)	0.897
*tst*	5(5.0)	3(12.5)	2(2.6)	0.157

### MRSA identification and Staphylococcus chromosomal cassette *mec* (SCC*mec*) typing

Among 101 *S. aureus* isolates, 24 (23.8%) were identified as MRSA. Three SCC*mec* types (II, III, and IVa) were identified in 20 (83.3%, 20/24) MRSA isolates, while the other four isolates (16.7%, 4/24) could not be typed. SCC*mec* IVa was the predominant type in MRSA isolates (54.2%, 13/24), while SCC*mec* III and SCC*mec* II were detected with lower frequencies (20.8 and 8.3%, respectively). It is worth noting that these two SCC*mec* types were only detected in *S. aureus* collected from 2016 to 2018. *S. aureus* isolated in years 2019 and 2020 were all grouped into SCC*mec* IVa, except for the four isolates that could not be typed.

### Multilocus sequence typing (MLST) profiles

**Fig. 1:**
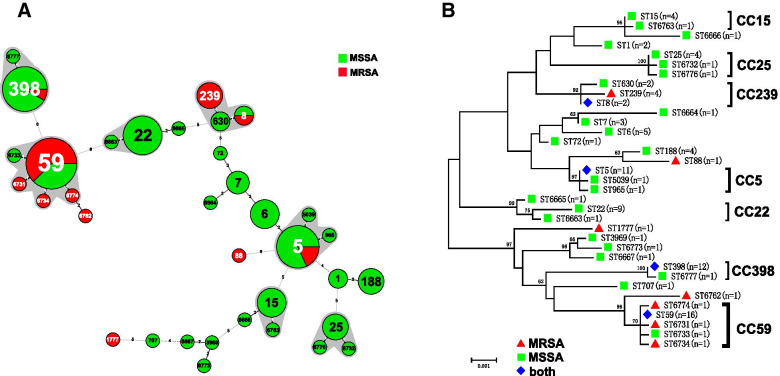
Phylogenetic and population structure analysis of ***Staphylococcus aureus*** isolates. **A** Minimum-spanning tree of 101 *S. aureus* isolates from blood based on MLST. Each ST is represented by a circle sized in proportion to the number of isolates represented by that ST. The gray shaded areas surrounding the STs denote types that belong to the same CC. The number of allelic differences between STs is indicated on the branches. MRSA isolates are represented by red color while MSSA isolates are represented by green color. The detailed MLST profiles can be seen in the Additional file 2. **B** The maximum likelihood tree of 101 *S. aureus* isolates for MLST typing. The maximum likelihood tree was constructed based on the seven combined housekeeping genes sequences. Bootstrap support was based on 1000 replicates, and only branch nodes higher than 60 were shown

Among the 101 *S. aureus* isolates, 20 existing STs and 15 novel STs were found (Fig. 1 and Additional file 2). The novel STs were submitted for ST assignment with the assigned numbers of ST6663 to ST6667, ST6731 to ST6734, ST6762 to ST6763, ST6773 to ST6774, and ST6776 to ST6777. The profiles of the newly identified ST types are listed in Table 3. In the alignment of MLST sequence, eight novel sequences were found in *arcC*, *glpF*, *tpi*, and *yqiL* each, which were designated as *arcC 790*, *arcC 794*, *glpF 862*, *glpF 864*, *tpi 757*, *tpi 762*, *yqiL 901*, and *yqiL 912.* Moreover, 78.2% (79/101) were represented by 13 main STs (having ≥2 isolates), and the prevalent STs were ST59, ST398, and ST5, accounting for 15.8% (16/101), 11.9% (12/101), and 10.9% (11/101), respectively (Fig. 1 and Additional file 2). Seven clonal complexes (CCs) and 15 singletons were identified by Bionumerics. CC59 (19.8%) was the most prevalent CC, followed by CC398 (12.9%) and CC5 (12.9%) (Fig. 1.A and Table 4). The phylogenetic tree analysis results are highly consistent with that of the minimum spanning tree. Isolates of the same clonal complex also belong to the same cluster on the phylogenetic tree (Fig. 1.B).

**Table 3:** Allelic profiles of the novel STs found in this study

ST	*arcC*	*aroE*	*glpF*	*gmk*	*pta*	*tpi*	*yqiL*
ST6663	7	6	1	5	19	8	6
ST6664	5	4	444	4	8	6	15
ST6665	7	6	1	5	7	8	3
ST6666	13	13	1	1	7	11	5
ST6667	6	5	6	2	7	14	13
ST6731	19	23	15	2	19	20	901
ST6732	4	1	4	1	5	757	4
ST6733	790	23	15	2	19	20	15
ST6734	19	23	862	2	19	20	15
ST6762	19	23	864	2	4	20	6
ST6763	794	13	1	1	12	11	13
ST6773	6	5	6	2	12	14	5
ST6774	19	23	864	2	19	20	15
ST6776	4	1	4	1	5	762	4
ST6777	3	35	19	2	20	26	912

**Table 4:** Molecular characteristics of *S. aureus* isolates collected in this study

CC(no.)	MLST(no.)	*Spa*(no.)	MRSA(no.)	MSSA(no.)	SCC*mec*(no.)
CC59(20)	ST59(16)	t437(12)	10	2	IVa(10)
		t163(2)		2	
		t1950(1)		1	
		t441(1)		1	
	ST6731(1)	t437(1)	1		IVa(1)
	ST6733(1)	t437(1)		1	
	ST6734(1)	t172(1)	1		IVa(1)
	ST6774(1)	t1751(1)	1		NT(1)
CC398(13)	ST398(12)	t571(9)	1	8	III(1)
		t034(2)		2	
		t7160(1)		1	
	ST6777(1)	NT(1)		1	
CC5(13)	ST5(11)	t002(4)		4	
		t954(2)		2	
		t1084(1)		1	
		t13506(1)		1	
		t2460(1)	1		II(1)
		t5076(1)	1		II(1)
		t668(1)		1	
	ST965(1)	t062(1)		1	
	ST5039(1)	t002(1)		1	
CC22(10)	ST22(9)	t309(5)		5	
		t1516(1)		1	
		t2336(1)		1	
		t5335(1)		1	
		t7611(1)		1	
	ST6663(1)	t5335(1)		1	
CC15(5)	ST15(4)	t084(4)		4	
		t14014(1)		1	
	ST6763(1)	t084(1)		1	
CC239(8)	ST239(4)	t030(3)	3		III(3)
		t037(1)	1		NT(1)
	ST630(2)	t377(2)		2	
	ST8(2)	t008(1)	1		IVa(1)
		t024(1)		1	
CC25(6)	ST25(4)	t349(1)		1	
		t078(1)		1	
		t472(1)		1	
		t078(1)		1	
	ST6732(1)	t18446(1)		1	
	ST6776(1)	NT(1)		1	
Singletons(25)	ST6(5)	t701(4)		4	
		t2467(1)		1	
	ST188(4)	t189(4)		4	
	ST7(3)	t796(2)		2	
		t091(1)		1	
	ST1(2)	t127(2)		2	
	ST72(1)	t324(1)		1	
	ST88(1)	t1764(1)	1		NT(1)
	ST707(1)	t4523(1)		1	
	ST1777(1)	t318(1)	1		III(1)
	ST3969(1)	t159(1)		1	
	ST6664(1)	t796(1)		1	
	ST6665(1)	t309(1)		1	
	ST6666(1)	t346(1)		1	
	ST6667(1)	t2091(1)		1	
	ST6762(1)	t1751(1)	1		NT(1)
	ST6773(1)	t2092(1)		1	

When ST types and oxacillin sensitivity were combined analysis, the five predominant genotypes were ST398-MSSA (10.9%), ST59-MRSA (9.9%), ST22-MSSA (8.9%), ST5-MSSA (8.9%), and ST59-MSSA (5.9%). When the STs and SCC*mec* typing were combined, the predominant combination was ST59-SCC*mec*IVa (41.7%, 10/24), followed by ST239-III (12.5%, 3/24) and ST5-II (8.3%, 2/24). Besides, there was a strong association between certain STs and SCC*mec* typing, with all the 10 ST59-MRSA isolates identified as SCC*mec* IVa, both of the two ST5-MRSA isolates as SCC*mec* II, and most (75%, 3/4) ST239-MRSA isolates as SCC*mec* III. When the analyses of ST types and MDR phenotypes were combined, we observed that the predominant MDR clone in MRSA was ST59 (43.5%, 10/23) followed by ST239 (17.4%, 4/23). Whereas ST5 (16.7%, 6/54) accounted for the majority of MDR MSSA isolates, followed by ST22 (13.0%, 7/54).

### Staphylococcal protein A (*Spa*) profiles

*Spa* analysis revealed a total of 49 different *spa* types in this study with two isolates not capable of being identified for *spa* (Table 4 and Additional file 2). The most prevalent *spa* type was t437 (13.5%) and t571 (8.7%). t571 was also the dominant *spa* type in samples obtained in 2016 and 2017. However, only one t571 isolate could be found in each year from 2018 to 2020 and the dominant ST was changed to t437. When ST and *spa* typing were combined, a strong association were observed between certain STs and *spa* types, with ST59 type primarily associated with t437 (75.0%, 12/16), ST398 with t571 (75.0%, 9/12), ST188 with t189 (100%, 5/5), ST15 with t084 (80%, 4/5) and ST16 with t701 (80%, 4/5). ST59-t437 was the most predominant combinations (11.9%, 12/101) among all of the 101 isolates in this study, followed by ST398-t571 (8.9%, 9/101). Other ST and *spa* types were represented by no more than four isolates per type.

### Characteristics of the major clones ST59-t437 and ST398-t571

When the analyses of ST types, *spa* types, and OXA sensitivity were combined, we observed that ST59-t437 and ST398-t571 were also the most abundant ST-*spa* types in MRSA and MSSA isolates. Among them, ST59-t437 was detected in 10 isolates from MRSA and only two isolates in MSSA. In contrast, eight ST398-t571 isolates belonged to MSSA and only one ST398-t571 isolate belonged to MRSA. Of the 15 antibiotics tested in this study, ST59-t437 isolates had significantly higher resistance rates than ST398-t571 isolates for penicillin, cefoxitin, oxacillin, erythromycin, clindamycin, and tetracycline (*P* < 0.05). No significant differences were found in the resistance to other antibiotics between ST59-t437 and ST398-t571 isolates (Table 5). Among the 17 virulence genes tested in this study, *seb* and *hlb* were more frequently seen in ST59-t437 isolates than in ST398-t571 isolates (*P* < 0.05). No significant differences were found in the positive rates for the remaining virulence genes between these two types of strains (Table 6).

**Table 5:** Resistance rates among ST59-t437 and ST398-t571 isolates

Antimicrobial	Resistance rate (%)		*P*-value
	ST59-t437	ST398-t571	
	(*n* = 12)	(*n* = 9)	
Penicillin	12(100.0%)	4(44.4%)	0.006
Cefoxitin	10(83.3%)	1(11.1%)	0.002
Oxacillin	10(83.3%)	1(11.1%)	0.002
Gentamicin	0(0.0%)	0(0.0%)	–
Ciprofloxacin	1(8.3%)	1(11.1%)	1.000
Levofloxacin	1(8.3%)	1(11.1%)	1.000
Moxifloxacin	1(8.3%)	1(11.1%)	1.000
Erythromycin	12(100.0%)	5(55.6%)	0.021
Clindamycin	12(100.0%)	5(55.6%)	0.021
Linezolid	0(0.0%)	0(0.0%)	–
Vancomycin	0(0.0%)	0(0.0%)	–
Tetracycline	7(58.3%)	0(0.0%)	0.007
Tigecycline	0(0.0%)	0(0.0%)	–
Rifampicin	0(0.0%)	0(0.0%)	–
Trimethoprim/Sulfamethoxazole	0(0.0%)	2(22.2%)	0.171

**Table 6:** Prevalence of virulence genes among ST59-t437 and ST398-t571 isolates

gene	distributing in		*P*-value
	ST59-t437 (*n* = 12); n (%)	ST398-t571 (*n* = 9); n (%)	
*eta*	1(8.3)	0(0)	1.000
*etb*	0(0)	0(0)	–
*sea*	0(0)	1(11.1)	0.429
*seb*	8(66.7)	0(0)	0.005
*sec*	1(8.3)	0(0)	1.000
*sed*	5(41.7)	3(33.3)	1.000
*see*	0(0)	0(0)	–
*seg*	3(25.0)	0(0)	0.229
*seh*	0(0)	0(0)	–
*sei*	3(25.0)	0(0)	0.229
*sej*	4(33.3)	3(33.3)	1.000
*hla*	12(100)	9(100)	–
*hlb*	12(100)	1(11.1)	0.000
*hld*	12(100)	9(100)	–
*hlg*	12(100)	9(100)	–
*pvl*	2(16.7)	1(11.1)	1.000
*tst*	0(0)	0(0)	–

## Discussion

Bloodstream infection of *S. aureus* can be life-threatening and present great obstacles for effective clinical treatments [21]. Our present study offers insights into the clonality, virulence genes, and antibiotic resistance of *S. aureus* from blood.

China antimicrobial surveillance network (CHINET) surveillance data have shown that from 2005 to 2020 the detection rate of MRSA isolates in China decreased year by year from 69.0 to 31.0% (www.chinets.com). Compared to these data, the resistance rate of *S. aureus* for oxacillin in our present study was 23.9%, which was roughly one-third of the rate reported in 2005 and 7% lower than the rate in 2020. For comparison, the prevalence of MRSA was 44% in the United States in 2016, 19% in Australia in 2017, 27% in South Africa in 2016, and 7% in the United Kingdom in 2017 (resistancemap.cddep.org). These results suggested that the prevalence of MRSA varies among different countries and provinces. In this study, we also found that 23 MRSA isolates and 54 MSSA isolates were resistant to multiple drugs and most of them showed co-resistance to penicillin, erythromycin, and clindamycin. In contrast, the resistance rate to rifampicin was only 3.0% and all isolates were sensitive to vancomycin, linezolid, and tigecycline. Our finding was similar to the results of the majority of studies conducted in China and worldwide [22–24], indicating that these drugs would be therapeutic agents to control infections.

Virulence genes can play a critical role in the pathogenicity of *S. aureus*. However, the distribution of these genes might differ in different strains. Therefore, detecting the distribution of virulence genes is valuable for epidemiological control of *S. aureus*. PVL can target neutrophils and often cause severe infection. A previous study in Urumqi showed that 80.8% of ST22-MSSA strains harbored the *pvl* gene [25]. Xiao et al. also found that 83.3% of ST22 isolates were *pvl* positive [26]. In the current study, a total of 22 *pvl* positive isolates were identified, and all the nine ST22 isolates contained this gene. Our results agree with previous findings that ST22 isolates tended to harbor *pvl* gene than other ST types [27], but the detailed molecular mechanism requires further investigation. Hemolysin is another important cytotoxic molecule in *S. aureus* which mainly acts on the erythrocyte. In the present study, *hla*, *hld*, and *hlg* genes were detected in all the *S. aureus* isolates, but *hlb* gene could only be detected in 37 isolates and showed different distributions in MRSA and MSSA strains. MRSA tended to carry *hlb* gene, which was comparable with some previous studies [20, 28]. *Hlgb* was the coding gene of β-hemolysin which was one type of hemolysin produced by *S. aureus*. By lysing erythrocytes, this virulence factor helps *S. aureus* evade the host immune system and scavenge nutrients [29], which could increase the survival of *S. aureus* in human blood [11]. Therefore, the higher detection rate of *hlb* gene may confer more MRSA isolates stronger survival ability in blood and contribute to the increased morbidity and longer hospital length of stay of MRSA BSI than those with MSSA BSI [10]. Enterotoxins are the major factor to cause food poisoning. These virulence factors are also regarded as superantigens and have profound effects on the immune system. Although the *sea* was found to be the most prevalent enterotoxin gene for *S. aureus* isolated from blood in China [18], only 13.9% *S. aureus* isolates harbored this gene in this study, while *seg* and *sei* were the predominant enterotoxin genes. Moreover, *seg* and *sei* were detected more frequently in MSSA isolates than in MRSA. These results might represent regional characteristics in Shandong, China.

MLST method has been widely used to depict molecular characteristics of isolates recovered from various geographic regions. Previous studies showed that in China, ST5 and ST59 were the most prevalent BSI MRSA STs in 2016 and 2018, respectively [19, 20]. These two genotypes were also the major epidemic clone of MRSA in East Asia [30]. Importantly, in China, the prevalence of ST59-MRSA among all MRSA isolates of BSIs increased from 4.5% [18] in 2010–2011 to 9.1% in 2013 and 19.8% in 2016 [19]. In the present study, although most of the ST5 isolates were MSSA and only one MRSA isolate belonged to ST5, we also found that ST59 was the most abundant ST type among all MRSA isolates. Taken together, these findings suggested that ST59 MRSA has emerged as an important invasive pathogen for bloodstream infections in China which should be taken seriously. ST398 was the dominant ST in MSSA in our study with a detection rate of 10.9%, which was comparable to the observations in China [19, 20] and France [31, 32] but different from those in Germany and Netherlands [33, 34]. When ST and *spa* types were combined, the most predominant genotypes were ST59-t437 and ST398-t571 in this study. Among them, ST398-t571 was the most prevalent pattern for *S. aureus* isolated from blood in 2016 and 2017. However, from 2018 to 2020, the dominant pattern was replaced by ST59-t437, and only one ST398-t571 isolate was found each year. Our finding was comparable to the observation in Li et al.’s study from 2013 to 2016 in China [19], which found a decreased detection rate of ST398-t571 from 2.7 to 0.9% and an increased detection rate of ST59-t437 from 5.5 to 6.4%. Besides, Li et al. also reported that ST59-t437 was the major cause of bloodstream infection in 2016 in China. Interestingly, in this study, we also found that ST59-t437 and ST398-t571 had different hemolysin patterns. All the ST59-t437 isolates were positive for *hlgb* gene, whereas most (88.9%) ST398-t571 bacteria did not contain this gene. Considering the important role of β-hemolysin in the survival of *S. aureus* in human blood, the deficiency of *hlgb* gene may lead to a lack of selective advantage of ST398-t571 isolates in human blood. Besides, a previous study performed by Yang et al. showed that ST59-t437 strain had a stronger biofilm-forming capacity than ST398 isolates [35]. As biofilm formation capacity could increase the ability of *S. aureus* to survive in the hospital environment and protect isolates from immune defenses [36, 37], the stronger biofilm formation capacity might contribute to the higher detection rate for bloodstream infection. Taken together, the existence of *hlgb* gene and the stronger biofilm formation capacity may confer some advantages for ST59-t437 strains for bloodstream infections. Further investigation is needed to reveal the molecular mechanism.

We also observed a total of 15 new STs in this study. Among them, one was classified into CC398, four into CC59, one into CC22, one into CC15, two into CC25, and others were singletons, which suggested that *S. aureus* isolates were diverse and still in clonal expansion. As four of the 15 isolates in the new STs were identified as MRSA, close attention should be paid toward these new STs to identify and further limit both transmission and outbreaks.

## Conclusions

In summary, we characterized the clonality, virulence genes, and antibiotic resistance of *S. aureus* from blood in a hospital in Shandong, China. A changing of the predominant genotype from ST398-t571 to ST59-t437 was found in this hospital. These two genotypes also have different antibiotic resistance and virulence genes profiles. Additionally, 15 novel STs were detected, and some of them were MRSA. Collectively, our findings offer new epidemiological data of blood *S. aureus* strains, which can help improve infection control measures and clinical treatments in hospitals.

## Methods

### Bacterial isolates

A total of 101 nonrepetitive *S. aureus* isolates were obtained from blood samples of patients from different departments (neurosurgery, intensive care unit (ICU), hematopathology, and other wards) at the First Affiliated Hospital of Shandong First Medical University (Shandong, China). These samples were obtained from 2016 to 2020. Blood specimens were collected from patients with suspected bacterial BSIs according to CDC criteria [38]. Blood cultures flagging positive were inoculate on Columbia blood agar, chocolate agar, and MacConkey agar. All agars were incubated at 37 °C for 24–48 h in 10% CO_2_. The suspected bacteria were identified using the VITEK-2 compact (BioMérieux, France) GP colorimetric identification card and further confirmed using matrix assisted laser desorption ionization time of flight mass spectrometry (MALDI-TOF MS) (Bruker).

### Antimicrobial susceptibility test

To test susceptibility, all *S. aureus* isolates were exposed to 15 antibiotics, including penicillin, cefoxitin, oxacillin, gentamicin, ciprofloxacin, levofloxacin, moxifloxacin, erythromycin, clindamycin, linezolid, vancomycin, tetracycline, tigecycline, rifampicin, and trimethoprim/sulfamethoxazole using Vitek 2 compact system (BioMérieux, France) with AST-GP-67 cards. The results were evaluated according to the Clinical and Laboratory Standards Institute (CLSI) criteria.

### Detection of virulence genes

To detect virulence genes in *S. aureus* isolates, polymerase chain reaction (PCR) assays were carried out using conventional PCR amplification [39, 40]. The target virulence genes included the exfoliative toxin genes (*eta* and *etb*), staphylococcal enterotoxin genes (*sea*-*see* and *seg*-*sej*), hemolysin genes (*hla*, *hlb*, *hld*, and *hlg*), the *pvl* gene (lukF/SPV), and the toxic shock syndrome toxin gene (*tst*). Positive amplicons were randomly selected for sequencing to verify the amplicons sequences.

### SCC*mec* typing

MRSA strains were confirmed by the presence of the *mecA* gene, and SCC*mec* classification was performed as previously described [41].

### *Spa* sequencing

*Spa* typing was performed based on the Ridom Staph Type standard protocol. Sequencing results were analyzed using BioNumerics software (version 7.6, Applied Maths), which automatically analyzes *spa* repeats and assigns *spa* types [42]. *S. aureus* isolates that could not be classified as any known *spa* type were defined as nontypable (NT).

### MLST

MLST analyses were performed for all *S. aureus* isolates as described previously [43]. The amplified fragments of seven housekeeping genes (*arcC*, *aroE*, *glpF*, *gmk*, *pta*, *tpi*, *yqiL*) were sequenced in both directions. The sequences were aligned with the reference sequence from the MLST database (https://pubmlst.org/saureus/). Newly identified STs were submitted to the MLST database curator for approval, and new numbers were assigned. A minimum-spanning tree using the allelic difference between isolates of the seven housekeeping genes was constructed using BioNumerics (version 7.6, Applied Maths) software. A maximum likelihood tree was constructed based on the seven combined housekeeping genes sequences using the MEGA software, and only one sequence from the same sequence types (STs) was used to avoid duplication.

### Statistical analysis

Statistical analyses were performed using SPSS Statistics 21.0 for Windows. The resistance rates and distributions of virulence genes between MRSA and MSSA as well as between ST59-t437 and ST398-t571 isolates were compared using the chi-square test. A two-sided *p*-value of less than 0.05 was considered statistically significant.

## Supplementary Information

**Additional file 1.**

**Additional file 2.**

## Data Availability

The datasets used and/or analyzed during the current study are within the manuscript and the Additional files.

## Abbreviations

BSI: Bloodstream infection
PCR: Polymerase chain reaction
MLST: Multilocus sequence typing
MRSA: Methicillin-resistant *Staphylococcus aureus*
MSSA: Methicillin-susceptible *Staphylococcus aureus*
ST: Sequence type
SCC*mec*: Staphylococcus chromosomal cassette *mec*
PVL: Panton-valentine leukocidin
TSST-1: Toxic shock syndrome toxin-1
ETs: Exfoliative toxins
SE: Staphylococcal enterotoxin
SSSS: Staphylococcal-scalded skin syndrome
OXA: Oxacillin
CC: Clonal complex
CLSI: Clinical and laboratory standards institute
CHINET: China antimicrobial surveillance network
ICU: Intensive care unit
MIC: Minimum inhibitory concentration
MALDI-TOF MS: Matrix assisted laser desorption ionization time of flight mass spectrometry
MDR: Multidrug resistant.

## References

[1] Dayan GH, Mohamed N, Scully IL, Cooper D, Begier E, Eiden J, Jansen KU, Gurtman A, Anderson AS (2016). Staphylococcus aureus: the current state of disease, pathophysiology and strategies for prevention. Expert Rev Vaccines.

[2] Lowy FD (1998). Staphylococcus aureus infections. N Engl J Med.

[3] López-Cortés LE, Gálvez-Acebal J, Rodríguez-Baño J (2020). Therapy of Staphylococcus aureus bacteremia: evidences and challenges. Enferm Infecc Microbiol Clin.

[4] Martinez RM, Wolk DM. Bloodstream infections. Microbiol Spectr. 2016;4(4):1–34. 10.1128/microbiolspec.DMIH2-0031-2016 27726765

[5] Kritikos A, Manuel O (2016). Bloodstream infections after solid-organ transplantation. Virulence.

[6] Mendes RE, Sader HS, Castanheira M, Flamm RK (2018). Distribution of main Gram-positive pathogens causing bloodstream infections in United States and European hospitals during the SENTRY Antimicrobial Surveillance Program (2010-2016): Concomitant analysis of oritavancin in vitro activity. J Chemother.

[7] Hu F, Guo Y, Yang Y, Zheng Y, Wu S, Jiang X, Zhu D, Wang F (2019). Resistance reported from China antimicrobial surveillance network (CHINET) in 2018. Eur J Clin Microbiol Infect Dis.

[8] Lakhundi S, Zhang K. Methicillin-resistant *Staphylococcus aureus*: molecular characterization, evolution, and epidemiology. Clin Microbiol Rev. 2018;31(4): 1–103. 10.1128/CMR.00020-18 PMC6148192 30209034

[9] Whitby M, McLaws ML, Berry G (2001). Risk of death from methicillin-resistant Staphylococcus aureus bacteraemia: a meta-analysis. Med J Aust.

[10] Rodrigues R, Passadouro R, Gomes O, Castro R (2020). Risk factors, length of stay and in-hospital mortality of methicillin-resistant Staphylococcus aureus infections: a Case-control study. Acta Med Port.

[11] Oliveira D, Borges A, Simões M. *Staphylococcus aureus* toxins and their molecular activity in infectious diseases. Toxins. 2018;10(6):1–19. 10.3390/toxins10060252 PMC6024779 29921792

[12] Mir Z, Nodeh Farahani N, Abbasian S, Alinejad F, Sattarzadeh M, Pouriran R, Dahmardehei M, Mirzaii M, Khoramrooz SS, Darban-Sarokhalil D (2019). The prevalence of exotoxins, adhesion, and biofilm-related genes in Staphylococcus aureus isolates from the main burn center of Tehran, Iran. Iran J Basic Med Sci.

[13] Salgado-Pabón W, Case-Cook LC, Schlievert PM (2014). Molecular analysis of staphylococcal superantigens. Methods Mol Biol.

[14] Bukowski M, Wladyka B, Dubin G (2010). Exfoliative toxins of Staphylococcus aureus. Toxins.

[15] O'Hara FP, Amrine-Madsen H, Mera RM, Brown ML, Close NM, Suaya JA, Acosta CJ (2012). Molecular characterization of Staphylococcus aureus in the United States 2004-2008 Reveals the rapid expansion of USA300 among inpatients and outpatients. Microb Drug Resist.

[16] Hughes J, Stabler R, Gaunt M, Karadag T, Desai N, Betley J, Ioannou A, Aryee A, Hearn P, Marbach H (2015). Clonal variation in high- and low-level phenotypic and genotypic mupirocin resistance of MRSA isolates in south-East London. J Antimicrob Chemother.

[17] Bouiller K, Bertrand X, Hocquet D, Chirouze C. Human infection of methicillin-susceptible *Staphylococcus aureus* CC398: a review. Microorganisms. 2020;8(11):1–19. 10.3390/microorganisms8111737 PMC7694499 33167581

[18] He W, Chen H, Zhao C, Zhang F, Li H, Wang Q, Wang X, Wang H (2013). Population structure and characterisation of Staphylococcus aureus from bacteraemia at multiple hospitals in China: association between antimicrobial resistance, toxin genes and genotypes. Int J Antimicrob Agents.

[19] Li S, Sun S, Yang C, Chen H, Yin Y, Li H, Zhao C, Wang H (2018). The changing pattern of population structure of Staphylococcus aureus from bacteremia in China from 2013 to 2016: ST239-030-MRSA replaced by ST59-t437. Front Microbiol.

[20] Li X, Fang F, Zhao J, Lou N, Li C, Huang T, Li Y (2018). Molecular characteristics and virulence gene profiles of Staphylococcus aureus causing bloodstream infection. Br J Infect Dis.

[21] Jung N, Rieg S (2018). Essentials in the management of S. aureus bloodstream infection. Infection.

[22] Fu Y, Xiong M, Li X, Zhou J, Xiao X, Fang F, Cheng X, Le Y, Li Y (2020). Molecular characteristics, antimicrobial resistance and virulence gene profiles of Staphylococcus aureus isolates from Wuhan, Central China. Infec Drug Resist.

[23] Gu F, He W, Xiao S, Wang S, Li X, Zeng Q, Ni Y, Han L (2020). Antimicrobial resistance and molecular epidemiology of Staphylococcus aureus causing bloodstream infections at Ruijin Hospital in Shanghai from 2013 to 2018. Sci Rep.

[24] Monaco M, Pimentel de Araujo F, Cruciani M, Coccia EM, Pantosti A (2017). Worldwide epidemiology and antibiotic resistance of Staphylococcus aureus. Curr Top Microbiol Immunol.

[25] Yuan W, Liu J, Zhan Y, Wang L, Jiang Y, Zhang Y, Sun N, Hou N (2019). Molecular typing revealed the emergence of pvl-positive sequence type 22 methicillin-susceptible Staphylococcus aureus in Urumqi, northwestern China. Infect Drug Resist.

[26] Xiao N, Yang J, Duan N, Lu B, Wang L (2019). Community-associated Staphylococcus aureus PVL(+) ST22 predominates in skin and soft tissue infections in Beijing, China. Infect Drug Resist.

[27] Chen Y, Liu Z, Duo L, Xiong J, Gong Y, Yang J, Wang Z, Wu X, Lu Z, Meng X (2014). Characterization of Staphylococcus aureus from distinct geographic locations in China: an increasing prevalence of spa-t030 and SCCmec type III. PLoS One.

[28] Liu Y, Du FL, Liu PP, Mei YF, Wan LG, Wei DD, Xu HY, Zhang W (2018). Molecular epidemiology and virulence features of Staphylococcus aureus bloodstream isolates in a regional burn Center in China, 2012-2016. Microb Drug Resist.

[29] Huseby M, Shi K, Brown CK, Digre J, Mengistu F, Seo KS, Bohach GA, Schlievert PM, Ohlendorf DH, Earhart CA (2007). Structure and biological activities of beta toxin from Staphylococcus aureus. J Bacteriol.

[30] Ward MJ, Goncheva M, Richardson E, McAdam PR, Raftis E, Kearns A, Daum RS, David MZ, Lauderdale TL, Edwards GF (2016). Identification of source and sink populations for the emergence and global spread of the East-Asia clone of community-associated MRSA. Genome Biol.

[31] Bonnet I, Millon B, Meugnier H, Vandenesch F, Maurin M, Pavese P, Boisset S (2018). High prevalence of spa type t571 among methicillin-susceptible Staphylococcus aureus from bacteremic patients in a French University hospital. PLoS One.

[32] Sauget M, Bouiller K, Richard M, Chagrot J, Cholley P, Hocquet D, Bertrand X (2019). Increasing incidence of bloodstream infections due to Staphylococcus aureus clonal complex 398 in a French hospital between 2010 and 2017. Eur J Clin Microbiol Infect Dis.

[33] Cuny C, Layer F, Köck R, Werner G, Witte W. Methicillin susceptible Staphylococcus aureus (MSSA) of clonal complex CC398, t571 from infections in humans are still rare in Germany. PLoS One. 2013, e83165;8(12). 10.1371/journal.pone.0083165 PMC3867410 24367584

[34] Verkade E, Bergmans AM, Budding AE, van Belkum A, Savelkoul P, Buiting AG, et al. Recent emergence of Staphylococcus aureus clonal complex 398 in human blood cultures. PLoS One. 2012, e41855;7(10). 10.1371/journal.pone.0041855 PMC3475701 23094014

[35] Yang X, Qian S, Yao K, Wang L, Liu Y, Dong F, Song W, Zhen J, Zhou W, Xu H (2017). Multiresistant ST59-SCCmec IV-t437 clone with strong biofilm-forming capacity was identified predominantly in MRSA isolated from Chinese children. BMC Infect Dis.

[36] Donlan RM, Costerton JW (2002). Biofilms: survival mechanisms of clinically relevant microorganisms. Clin Microbiol Rev.

[37] Lister JL, Horswill AR (2014). Staphylococcus aureus biofilms: recent developments in biofilm dispersal. Front Cell Infect Microbiol.

[38] Garner JS, Jarvis WR, Emori TG, Horan TC, Hughes JM (1988). CDC definitions for nosocomial infections, 1988. Am J Infect Control.

[39] Gu FF, Han LZ, Chen X, Wang YC, Shen H, Wang JQ, Tang J, Zhang J, Ni YX (2015). Molecular characterization of Staphylococcus aureus from surgical site infections in orthopedic patients in an orthopedic trauma clinical medical center in Shanghai. Surg Infect.

[40] Argudín MA, Tenhagen BA, Fetsch A, Sachsenröder J, Käsbohrer A, Schroeter A, Hammerl JA, Hertwig S, Helmuth R, Bräunig J (2011). Virulence and resistance determinants of German Staphylococcus aureus ST398 isolates from nonhuman sources. Appl Environ Microbiol.

[41] Zhang K, McClure JA, Elsayed S, Louie T, Conly JM (2005). Novel multiplex PCR assay for characterization and concomitant subtyping of staphylococcal cassette chromosome mec types I to V in methicillin-resistant Staphylococcus aureus. J Clin Microbiol.

[42] Li V, Chui L, Simmonds K, Nguyen T, Golding GR, Yacoub W, Ferrato C, Louie M (2014). Emergence of new CMRSA7/USA400 methicillin-resistant Staphylococcus aureus spa types in Alberta, Canada, from 2005 to 2012. J Clin Microbiol.

[43] Chen X, Wang WK, Han LZ, Liu Y, Zhang H, Tang J, Liu QZ, Huangfu YC, Ni YX (2013). Epidemiological and genetic diversity of Staphylococcus aureus causing bloodstream infection in Shanghai, 2009-2011. PLoS One.

