# Supplementary material for: Clonality, virulence genes, and antibiotic resistance of Staphylococcus aureus isolated from blood in Shandong, China
Source: BMC Microbiol. 2021 Oct 18;21:281. doi: 10.1186/s12866-021-02344-6 (PMC8522240; doi:10.1186/s12866-021-02344-6)
Supplement: Supplementary file 1 — Additional file 1. [file 12866_2021_2344_MOESM1_ESM.docx]

Table S1 Minimum Inhibitory Concentrations (MICs) of the tested antimicrobial agents

FOX: Cefoxitin; PEN: Penicillin; OXA: Oxacillin; GEN: Gentamicin; CIP: Ciprofloxacin; LEV: Levofloxacin; MFX: Moxifloxacin; ICR: Inducible clindamycin resistance; ERY: Erythromycin; CLI: Clindamycin; LZD: Linezolid; VAN: Vancomycin; TCY: Tetracycline; TGC: Tigecycline; RIF: Rifampicin; SXT: Trimethoprim/Sulfamethoxazole.

| **Strain** | **FOX** | **PEN** | **OXA** | **GEN** | **CIP** | **LEV** | **MFX** | **ICR** | **ERY** | **CLI** | **LZD** | **VAN** | **TCY** | **TGC** | **RIF** | **SXT** |
| --- | --- | --- | --- | --- | --- | --- | --- | --- | --- | --- | --- | --- | --- | --- | --- | --- |
| SA001 | - | ≥ 0.5 | 0.5 | ≤ 0.5 | ≤ 0.5 | 0.25 | ≤ 0.25 | + | ≥ 8 | ≤ 0.25 | 2 | 1 | ≤ 1 | ≤ 0.12 | ≤ 0.5 | 80 |
| SA002 | - | ≤ 0.03 | ≤ 0.25 | ≤ 0.5 | ≤ 0.5 | ≤ 0.12 | ≤ 0.25 | - | ≤ 0.25 | ≤ 0.25 | 2 | ≤ 0.5 | ≤ 1 | ≤ 0.12 | ≤ 0.5 | ≤ 10 |
| SA003 | - | ≥ 0.5 | ≤ 0.25 | ≤ 0.5 | ≥ 8 | 4 | 2 | - | ≥ 8 | ≥ 8 | 1 | ≤ 0.5 | ≤ 1 | ≤ 0.12 | ≤ 0.5 | ≤ 10 |
| SA004 | - | ≥ 0.5 | 0.5 | ≤ 0.5 | ≤ 0.5 | 0.25 | ≤ 0.25 | - | ≥ 8 | ≥ 8 | 2 | ≤ 0.5 | ≤ 1 | ≤ 0.12 | ≤ 0.5 | ≤ 10 |
| SA005 | - | ≥ 0.5 | 0.5 | ≤ 0.5 | 2 | 1 | ≤ 0.25 | - | ≥ 8 | ≤ 0.25 | 2 | 1 | ≤ 1 | ≤ 0.12 | ≤ 0.5 | ≤ 10 |
| SA006 | - | 0.06 | ≤ 0.25 | ≤ 0.5 | ≤ 0.5 | 0.25 | ≤ 0.25 | - | ≤ 0.25 | ≤ 0.25 | 2 | ≤ 0.5 | ≤ 1 | ≤ 0.12 | ≤ 0.5 | ≤ 10 |
| SA007 | - | ≥ 0.5 | 1 | ≤ 0.5 | ≤ 0.5 | 0.25 | ≤ 0.25 | + | ≥ 8 | ≤ 0.25 | 1 | ≤ 0.5 | ≥ 16 | ≤ 0.12 | ≤ 0.5 | ≤ 10 |
| SA008 | + | ≥ 0.5 | ≥ 4 | ≤ 0.5 | ≤ 0.5 | ≤ 0.12 | ≤ 0.25 | - | ≥ 8 | ≥ 8 | 2 | 1 | 2 | ≤ 0.12 | ≤ 0.5 | 160 |
| SA009 | + | ≥ 0.5 | ≥ 4 | ≤ 0.5 | 2 | 1 | ≤ 0.25 | - | ≥ 8 | ≥ 8 | 1 | ≤ 0.5 | ≤ 1 | ≤ 0.12 | ≤ 0.5 | ≤ 10 |
| SA010 | - | ≤ 0.03 | ≤ 0.25 | 2 | ≤ 0.5 | 0.25 | ≤ 0.25 | + | ≥ 8 | ≤ 0.25 | 2 | ≤ 0.5 | ≤ 1 | ≤ 0.12 | ≤ 0.5 | ≤ 10 |
| SA011 | - | 0.12 | ≤ 0.25 | 8 | ≤ 0.5 | ≤ 0.12 | ≤ 0.25 | - | ≥ 8 | ≥ 8 | 2 | ≤ 0.5 | ≤ 1 | ≤ 0.12 | ≤ 0.5 | 80 |
| SA012 | + | ≥ 0.5 | ≥ 4 | ≥ 16 | ≥ 8 | ≥ 8 | 4 | - | ≥ 8 | ≥ 8 | 2 | ≤ 0.5 | ≥ 16 | ≤ 0.12 | ≥ 32 | ≤ 10 |
| SA013 | - | ≥ 0.5 | 0.5 | ≤ 0.5 | ≤ 0.5 | 0.25 | ≤ 0.25 | + | ≥ 8 | ≤ 0.25 | 2 | ≤ 0.5 | ≤ 1 | ≤ 0.12 | ≤ 0.5 | ≤ 10 |
| SA014 | - | ≥ 0.5 | 0.5 | ≤ 0.5 | ≥ 8 | 4 | 2 | - | ≥ 8 | ≥ 8 | 2 | ≤ 0.5 | ≥ 16 | ≤ 0.12 | ≤ 0.5 | ≤ 10 |
| SA015 | - | ≥ 0.5 | ≤ 0.25 | ≤ 0.5 | 1 | 1 | ≤ 0.25 | + | ≥ 8 | ≤ 0.25 | 2 | 1 | ≤ 1 | ≤ 0.12 | ≤ 0.5 | ≤ 10 |
| SA016 | - | ≥ 0.5 | ≤ 0.25 | ≤ 0.5 | 1 | 0.5 | ≤ 0.25 | + | ≥ 8 | ≤ 0.25 | 2 | 1 | ≤ 1 | ≤ 0.12 | ≤ 0.5 | ≤ 10 |
| SA017 | - | ≥ 0.5 | ≤ 0.25 | ≤ 0.5 | ≤ 0.5 | ≤ 0.12 | ≤ 0.25 | + | ≥ 8 | ≤ 0.25 | 2 | ≤ 0.5 | ≤ 1 | ≤ 0.12 | ≤ 0.5 | ≤ 10 |
| SA018 | - | ≤ 0.03 | ≤ 0.25 | ≤ 0.5 | ≥ 8 | 4 | 1 | + | ≥ 8 | ≤ 0.25 | 1 | ≤ 0.5 | ≤ 1 | ≤ 0.12 | ≤ 0.5 | ≤ 10 |
| SA019 | - | ≥ 0.5 | 0.5 | ≤ 0.5 | ≤ 0.5 | ≤ 0.12 | ≤ 0.25 | - | ≥ 8 | ≥ 8 | 1 | ≤ 0.5 | ≤ 1 | ≤ 0.12 | ≤ 0.5 | ≤ 10 |
| SA020 | + | ≥ 0.5 | ≥ 4 | ≥ 16 | ≥ 8 | ≥ 8 | ≥ 8 | - | ≥ 8 | ≥ 8 | 2 | 1 | ≥ 16 | ≤ 0.12 | ≥ 32 | ≤ 10 |
| SA021 | - | ≥ 0.5 | ≤ 0.25 | ≤ 0.5 | ≤ 0.5 | 0.25 | ≤ 0.25 | + | ≥ 8 | ≤ 0.25 | 2 | ≤ 0.5 | ≤ 1 | ≤ 0.12 | ≤ 0.5 | ≤ 10 |
| SA022 | - | 0.06 | ≤ 0.25 | ≥ 16 | 2 | 1 | ≤ 0.25 | - | ≥ 8 | ≥ 8 | 2 | 1 | ≤ 1 | ≤ 0.12 | ≤ 0.5 | ≥ 320 |
| SA023 | - | 0.06 | ≤ 0.25 | ≤ 0.5 | ≤ 0.5 | 0.25 | ≤ 0.25 | - | ≤ 0.25 | ≤ 0.25 | 2 | ≤ 0.5 | ≤ 1 | ≤ 0.12 | ≤ 0.5 | ≤ 10 |
| SA024 | + | ≥ 0.5 | ≥ 4 | ≥ 16 | ≥ 8 | ≥ 8 | ≥ 8 | - | ≥ 8 | ≥ 8 | 2 | 1 | ≥ 16 | ≤ 0.12 | ≥ 32 | ≤ 10 |
| SA025 | - | ≤ 0.03 | ≤ 0.25 | ≤ 0.5 | ≤ 0.5 | 0.25 | ≤ 0.25 | - | ≤ 0.25 | ≤ 0.25 | 2 | 1 | ≤ 1 | ≤ 0.12 | ≤ 0.5 | ≤ 10 |
| SA026 | + | ≥ 0.5 | ≥ 4 | ≤ 0.5 | ≤ 0.5 | 0.25 | ≤ 0.25 | - | ≥ 8 | ≥ 8 | 2 | 1 | ≤ 1 | ≤ 0.12 | ≤ 0.5 | ≤ 10 |
| SA027 | - | ≥ 0.5 | ≤ 0.25 | 8 | ≥ 8 | 4 | 2 | - | ≥ 8 | ≥ 8 | 2 | 1 | ≥ 16 | ≤ 0.12 | ≤ 0.5 | ≤ 10 |
| SA028 | - | ≥ 0.5 | ≤ 0.25 | ≤ 0.5 | ≤ 0.5 | ≤ 0.12 | ≤ 0.25 | + | ≥ 8 | ≤ 0.25 | 1 | ≤ 0.5 | ≤ 1 | ≤ 0.12 | ≤ 0.5 | ≤ 10 |
| SA029 | - | ≥ 0.5 | ≤ 0.25 | ≤ 0.5 | ≤ 0.5 | ≤ 0.12 | ≤ 0.25 | - | ≤ 0.25 | ≤ 0.25 | 2 | 1 | ≤ 1 | ≤ 0.12 | ≤ 0.5 | ≤ 10 |
| SA030 | - | 0.25 | ≤ 0.25 | ≥ 16 | ≥ 8 | 4 | 2 | - | ≥ 8 | ≥ 8 | 2 | ≤ 0.5 | ≤ 1 | ≤ 0.12 | ≤ 0.5 | ≥ 320 |
| SA031 | - | ≥ 0.5 | 1 | ≥ 16 | ≤ 0.5 | ≤ 0.12 | ≤ 0.25 | - | ≥ 8 | ≥ 8 | 1 | ≤ 0.5 | ≥ 16 | ≤ 0.12 | ≤ 0.5 | 80 |
| SA032 | - | ≥ 0.5 | 0.5 | ≤ 0.5 | ≥ 8 | 4 | 2 | - | ≤ 0.25 | ≤ 0.25 | 2 | ≤ 0.5 | ≤ 1 | ≤ 0.12 | ≤ 0.5 | ≤ 10 |
| SA033 | + | ≥ 0.5 | ≥ 4 | ≤ 0.5 | ≤ 0.5 | 0.25 | ≤ 0.25 | - | ≥ 8 | ≥ 8 | 2 | ≤ 0.5 | ≥ 16 | ≤ 0.12 | ≤ 0.5 | ≤ 10 |
| SA034 | + | ≥ 0.5 | ≥ 4 | ≤ 0.5 | ≥ 8 | 4 | 2 | - | ≥ 8 | ≥ 8 | 1 | 1 | ≥ 16 | ≤ 0.12 | ≤ 0.5 | ≤ 10 |
| SA035 | - | ≥ 0.5 | 0.5 | ≤ 0.5 | ≤ 0.5 | ≤ 0.12 | ≤ 0.25 | + | ≥ 8 | ≤ 0.25 | 2 | 1 | ≤ 1 | ≤ 0.12 | ≤ 0.5 | ≤ 10 |
| SA036 | - | ≥ 0.5 | 0.5 | ≤ 0.5 | ≤ 0.5 | ≤ 0.12 | ≤ 0.25 | + | ≥ 8 | ≤ 0.25 | 2 | 1 | ≤ 1 | ≤ 0.12 | ≤ 0.5 | ≤ 10 |
| SA037 | - | 0.12 | ≤ 0.25 | ≤ 0.5 | ≤ 0.5 | 0.25 | ≤ 0.25 | + | ≥ 8 | ≤ 0.25 | 1 | ≤ 0.5 | ≤ 1 | ≤ 0.12 | ≤ 0.5 | ≤ 10 |
| SA038 | - | ≥ 0.5 | 0.5 | ≤ 0.5 | ≤ 0.5 | ≤ 0.12 | ≤ 0.25 | - | ≤ 0.25 | ≤ 0.25 | 2 | ≤ 0.5 | ≤ 1 | ≤ 0.12 | ≤ 0.5 | ≤ 10 |
| SA039 | + | ≥ 0.5 | ≥ 4 | ≤ 0.5 | ≥ 8 | ≥ 8 | 4 | + | ≥ 8 | ≤ 0.25 | 2 | 1 | ≥ 16 | ≤ 0.12 | ≤ 0.5 | ≤ 10 |
| SA040 | - | ≥ 0.5 | 0.5 | ≤ 0.5 | 1 | 1 | ≤ 0.25 | + | ≥ 8 | ≤ 0.25 | 2 | ≤ 0.5 | ≤ 1 | ≤ 0.12 | ≤ 0.5 | ≤ 10 |
| SA041 | - | ≥ 0.5 | ≤ 0.25 | ≤ 0.5 | ≥ 8 | 4 | 2 | - | ≤ 0.25 | ≤ 0.25 | 2 | ≤ 0.5 | ≤ 1 | ≤ 0.12 | ≤ 0.5 | ≤ 10 |
| SA042 | - | ≥ 0.5 | ≤ 0.25 | ≤ 0.5 | ≤ 0.5 | 0.25 | ≤ 0.25 | - | ≤ 0.25 | ≤ 0.25 | 2 | ≤ 0.5 | ≤ 1 | ≤ 0.12 | ≤ 0.5 | ≤ 10 |
| SA043 | - | ≥ 0.5 | ≤ 0.25 | ≤ 0.5 | 1 | 1 | ≤ 0.25 | + | ≥ 8 | ≤ 0.25 | 2 | 1 | ≤ 1 | ≤ 0.12 | ≤ 0.5 | ≤ 10 |
| SA044 | + | ≥ 0.5 | ≥ 4 | ≤ 0.5 | ≤ 0.5 | ≤ 0.12 | ≤ 0.25 | - | ≥ 8 | ≥ 8 | 1 | ≤ 0.5 | ≤ 1 | ≤ 0.12 | ≤ 0.5 | ≤ 10 |
| SA045 | - | ≥ 0.5 | 0.5 | ≤ 0.5 | ≤ 0.5 | ≤ 0.12 | ≤ 0.25 | + | ≥ 8 | ≤ 0.25 | 2 | ≤ 0.5 | ≤ 1 | ≤ 0.12 | ≤ 0.5 | ≤ 10 |
| SA046 | - | ≥ 0.5 | 0.5 | ≤ 0.5 | ≤ 0.5 | 0.25 | ≤ 0.25 | + | ≥ 8 | ≤ 0.25 | 2 | ≤ 0.5 | ≤ 1 | ≤ 0.12 | ≤ 0.5 | ≤ 10 |
| SA047 | - | ≥ 0.5 | ≤ 0.25 | ≥ 16 | 2 | 1 | ≤ 0.25 | - | ≥ 8 | ≥ 8 | 2 | 1 | ≤ 1 | ≤ 0.12 | ≤ 0.5 | ≥ 320 |
| SA048 | - | ≥ 0.5 | 0.5 | ≤ 0.5 | ≤ 0.5 | ≤ 0.12 | ≤ 0.25 | + | ≥ 8 | ≤ 0.25 | 2 | ≤ 0.5 | ≤ 1 | ≤ 0.12 | 2 | ≤ 10 |
| SA049 | + | ≥ 0.5 | ≥ 4 | ≤ 0.5 | ≤ 0.5 | 0.25 | ≤ 0.25 | - | ≥ 8 | ≥ 8 | 2 | ≤ 0.5 | ≥ 16 | ≤ 0.12 | ≤ 0.5 | ≤ 10 |
| SA050 | - | 0.25 | ≤ 0.25 | ≤ 0.5 | ≤ 0.5 | 0.25 | ≤ 0.25 | + | ≥ 8 | ≤ 0.25 | 2 | ≤ 0.5 | ≤ 1 | ≤ 0.12 | ≤ 0.5 | 160 |
| SA051 | - | ≥ 0.5 | 0.5 | ≤ 0.5 | ≤ 0.5 | 0.25 | ≤ 0.25 | + | ≥ 8 | ≤ 0.25 | 2 | ≤ 0.5 | ≤ 1 | ≤ 0.12 | ≤ 0.5 | ≤ 10 |
| SA052 | - | ≥ 0.5 | 0.5 | ≤ 0.5 | ≤ 0.5 | ≤ 0.12 | ≤ 0.25 | - | ≥ 8 | ≥ 8 | 2 | ≤ 0.5 | ≥ 16 | ≤ 0.12 | ≤ 0.5 | ≤ 10 |
| SA053 | + | ≥ 0.5 | ≥ 4 | ≥ 16 | ≥ 8 | ≥ 8 | 4 | - | ≥ 8 | ≥ 8 | 2 | ≤ 0.5 | ≥ 16 | ≤ 0.12 | ≤ 0.5 | ≤ 10 |
| SA054 | - | ≥ 0.5 | 2 | ≤ 0.5 | 1 | 1 | ≤ 0.25 | + | ≥ 8 | ≤ 0.25 | 1 | ≤ 0.5 | ≤ 1 | ≤ 0.12 | ≤ 0.5 | ≤ 10 |
| SA055 | + | ≥ 0.5 | ≥ 4 | ≤ 0.5 | ≤ 0.5 | 0.25 | ≤ 0.25 | - | ≥ 8 | ≥ 8 | 2 | ≤ 0.5 | ≥ 16 | ≤ 0.12 | ≤ 0.5 | ≤ 10 |
| SA056 | - | ≥ 0.5 | 0.5 | ≤ 0.5 | ≤ 0.5 | ≤ 0.12 | ≤ 0.25 | - | ≤ 0.25 | ≤ 0.25 | 2 | 1 | ≤ 1 | ≤ 0.12 | ≤ 0.5 | ≤ 10 |
| SA057 | + | ≥ 0.5 | ≥ 4 | ≤ 0.5 | ≤ 0.5 | ≤ 0.12 | ≤ 0.25 | - | ≥ 8 | ≥ 8 | 1 | 1 | ≤ 1 | ≤ 0.12 | ≤ 0.5 | ≤ 10 |
| SA058 | - | ≥ 0.5 | 0.5 | ≥ 16 | ≤ 0.5 | ≤ 0.12 | ≤ 0.25 | - | ≥ 8 | ≥ 8 | 2 | 1 | ≤ 1 | ≤ 0.12 | ≤ 0.5 | 80 |
| SA059 | - | ≥ 0.5 | 0.5 | ≤ 0.5 | ≤ 0.5 | ≤ 0.12 | ≤ 0.25 | + | ≥ 8 | ≤ 0.25 | 2 | ≤ 0.5 | ≤ 1 | ≤ 0.12 | ≤ 0.5 | ≤ 10 |
| SA060 | - | 0.06 | ≤ 0.25 | ≤ 0.5 | ≤ 0.5 | ≤ 0.12 | ≤ 0.25 | - | ≥ 8 | ≥ 8 | 2 | ≤ 0.5 | ≤ 1 | ≤ 0.12 | ≤ 0.5 | ≥ 320 |
| SA061 | - | ≥ 0.5 | ≤ 0.25 | 8 | ≤ 0.5 | ≤ 0.12 | ≤ 0.25 | - | ≥ 8 | ≤ 0.25 | 2 | ≤ 0.5 | ≥ 16 | ≤ 0.12 | ≤ 0.5 | ≤ 10 |
| SA062 | - | ≥ 0.5 | 0.5 | ≤ 0.5 | ≤ 0.5 | 0.25 | ≤ 0.25 | - | ≤ 0.25 | ≤ 0.25 | 2 | ≤ 0.5 | ≤ 1 | ≤ 0.12 | ≤ 0.5 | ≤ 10 |
| SA063 | - | ≥ 0.5 | 0.5 | ≤ 0.5 | ≥ 8 | 4 | 2 | + | ≥ 8 | ≤ 0.25 | 2 | 1 | ≤ 1 | ≤ 0.12 | ≤ 0.5 | ≤ 10 |
| SA064 | + | ≥ 0.5 | ≥ 4 | ≤ 0.5 | 2 | 1 | ≤ 0.25 | - | ≥ 8 | ≥ 8 | 1 | ≤ 0.5 | ≥ 16 | ≤ 0.12 | ≤ 0.5 | ≤ 10 |
| SA065 | + | ≥ 0.5 | ≥ 4 | ≤ 0.5 | ≤ 0.5 | ≤ 0.12 | ≤ 0.25 | - | ≤ 0.25 | ≤ 0.25 | 2 | 1 | ≤ 1 | ≤ 0.12 | ≤ 0.5 | ≤ 10 |
| SA066 | - | ≥ 0.5 | ≤ 0.25 | ≤ 0.5 | ≤ 0.5 | 0.25 | ≤ 0.25 | + | ≥ 8 | ≤ 0.25 | 2 | ≤ 0.5 | ≤ 1 | ≤ 0.12 | ≤ 0.5 | ≤ 10 |
| SA067 | + | ≥ 0.5 | ≥ 4 | 8 | ≥ 8 | 4 | 1 | - | ≥ 8 | ≤ 0.25 | 1 | ≤ 0.5 | ≥ 16 | ≤ 0.12 | ≤ 0.5 | ≤ 10 |
| SA068 | - | 0.25 | ≤ 0.25 | 8 | ≤ 0.5 | ≤ 0.12 | ≤ 0.25 | - | ≥ 8 | ≥ 8 | 2 | 1 | ≤ 1 | ≤ 0.12 | ≤ 0.5 | 160 |
| SA069 | - | ≥ 0.5 | 0.5 | ≤ 0.5 | ≥ 8 | 4 | 1 | + | ≥ 8 | ≤ 0.25 | 2 | 1 | ≤ 1 | ≤ 0.12 | ≤ 0.5 | 160 |
| SA070 | - | ≥ 0.5 | ≤ 0.25 | ≤ 0.5 | ≤ 0.5 | ≤ 0.12 | ≤ 0.25 | - | ≤ 0.25 | ≤ 0.25 | 2 | 1 | ≤ 1 | ≤ 0.12 | ≤ 0.5 | ≤ 10 |
| SA071 | - | ≥ 0.5 | 0.5 | 8 | ≤ 0.5 | 0.5 | ≤ 0.25 | - | ≥ 8 | ≥ 8 | 2 | 1 | ≤ 1 | ≤ 0.12 | ≤ 0.5 | ≥ 320 |
| SA072 | - | ≥ 0.5 | ≤ 0.25 | ≤ 0.5 | 4 | 4 | 1 | - | ≤ 0.25 | ≤ 0.25 | 2 | 1 | ≤ 1 | ≤ 0.12 | ≤ 0.5 | 80 |
| SA073 | - | ≥ 0.5 | ≤ 0.25 | ≤ 0.5 | ≤ 0.5 | ≤ 0.12 | ≤ 0.25 | - | ≤ 0.25 | ≤ 0.25 | 2 | ≤ 0.5 | ≤ 1 | ≤ 0.12 | ≤ 0.5 | ≤ 10 |
| SA074 | + | ≥ 0.5 | ≥ 4 | ≥ 16 | ≥ 8 | ≥ 8 | 4 | - | ≥ 8 | ≥ 8 | 2 | 1 | ≥ 16 | ≤ 0.12 | 2 | ≥ 320 |
| SA075 | - | ≥ 0.5 | ≤ 0.25 | ≥ 16 | 2 | 1 | ≤ 0.25 | - | ≥ 8 | ≥ 8 | 2 | ≤ 0.5 | ≤ 1 | ≤ 0.12 | ≤ 0.5 | ≥ 320 |
| SA076 | + | ≥ 0.5 | ≥ 4 | ≤ 0.5 | ≤ 0.5 | ≤ 0.12 | ≤ 0.25 | - | ≥ 8 | ≥ 8 | 1 | 1 | ≥ 16 | ≤ 0.12 | ≤ 0.5 | ≤ 10 |
| SA077 | - | ≥ 0.5 | ≤ 0.25 | ≤ 0.5 | ≤ 0.5 | 0.25 | ≤ 0.25 | - | ≤ 0.25 | ≤ 0.25 | 2 | 1 | ≤ 1 | ≤ 0.12 | ≤ 0.5 | ≤ 10 |
| SA078 | - | 0.12 | ≤ 0.25 | ≤ 0.5 | ≤ 0.5 | ≤ 0.12 | ≤ 0.25 | - | ≥ 8 | ≥ 8 | 2 | 1 | ≤ 1 | ≤ 0.12 | ≤ 0.5 | ≤ 10 |
| SA079 | + | ≥ 0.5 | ≥ 4 | ≤ 0.5 | ≤ 0.5 | 0.25 | ≤ 0.25 | - | ≥ 8 | ≥ 8 | 2 | 1 | ≤ 1 | ≤ 0.12 | ≤ 0.5 | ≤ 10 |
| SA080 | - | ≥ 0.5 | ≤ 0.25 | ≤ 0.5 | ≥ 8 | ≥ 8 | 2 | - | ≥ 8 | ≥ 8 | 2 | ≤ 0.5 | ≤ 1 | ≤ 0.12 | ≤ 0.5 | 80 |
| SA081 | - | 0.06 | ≤ 0.25 | ≤ 0.5 | ≤ 0.5 | 0.25 | ≤ 0.25 | - | ≤ 0.25 | ≤ 0.25 | 2 | ≤ 0.5 | ≤ 1 | ≤ 0.12 | ≤ 0.5 | ≤ 10 |
| SA082 | + | ≥ 0.5 | ≥ 4 | ≤ 0.5 | 2 | 1 | ≤ 0.25 | - | ≥ 8 | ≥ 8 | 2 | ≤ 0.5 | ≤ 1 | ≤ 0.12 | ≤ 0.5 | ≤ 10 |
| SA083 | - | ≥ 0.5 | ≤ 0.25 | ≤ 0.5 | ≤ 0.5 | ≤ 0.12 | ≤ 0.25 | + | ≥ 8 | ≤ 0.25 | 1 | 1 | ≤ 1 | ≤ 0.12 | ≤ 0.5 | ≤ 10 |
| SA084 | - | ≥ 0.5 | 0.5 | ≤ 0.5 | ≤ 0.5 | ≤ 0.12 | ≤ 0.25 | + | ≥ 8 | ≤ 0.25 | 1 | ≤ 0.5 | ≤ 1 | ≤ 0.12 | ≤ 0.5 | ≤ 10 |
| SA085 | - | ≥ 0.5 | 0.5 | ≤ 0.5 | ≤ 0.5 | ≤ 0.12 | ≤ 0.25 | + | ≥ 8 | ≤ 0.25 | 1 | ≤ 0.5 | ≤ 1 | ≤ 0.12 | ≤ 0.5 | ≤ 10 |
| SA086 | - | 0.12 | ≤ 0.25 | ≤ 0.5 | ≤ 0.5 | 0.25 | ≤ 0.25 | + | ≥ 8 | ≤ 0.25 | 1 | ≤ 0.5 | ≤ 1 | ≤ 0.12 | ≤ 0.5 | ≤ 10 |
| SA087 | - | ≥ 0.5 | 0.5 | ≥ 16 | ≥ 8 | 4 | 2 | - | ≥ 8 | ≥ 8 | 2 | ≤ 0.5 | ≤ 1 | ≤ 0.12 | ≤ 0.5 | ≥ 320 |
| SA088 | - | 0.06 | ≤ 0.25 | ≤ 0.5 | ≤ 0.5 | ≤ 0.12 | ≤ 0.25 | + | ≥ 8 | ≤ 0.25 | 2 | ≤ 0.5 | ≤ 1 | ≤ 0.12 | ≤ 0.5 | ≤ 10 |
| SA089 | - | ≥ 0.5 | ≤ 0.25 | 8 | ≥ 8 | 4 | 2 | - | ≥ 8 | ≥ 8 | 2 | ≤ 0.5 | ≤ 1 | ≤ 0.12 | ≤ 0.5 | 160 |
| SA090 | - | ≥ 0.5 | ≤ 0.25 | ≤ 0.5 | ≤ 0.5 | 0.25 | ≤ 0.25 | + | ≥ 8 | ≤ 0.25 | 2 | ≤ 0.5 | ≤ 1 | ≤ 0.12 | ≤ 0.5 | ≤ 10 |
| SA091 | + | ≥ 0.5 | ≥ 4 | ≤ 0.5 | ≤ 0.5 | 0.25 | ≤ 0.25 | - | ≥ 8 | ≥ 8 | 2 | 1 | ≥ 16 | ≤ 0.12 | ≤ 0.5 | ≤ 10 |
| SA092 | + | ≥ 0.5 | ≥ 4 | ≤ 0.5 | ≤ 0.5 | 0.25 | ≤ 0.25 | - | ≥ 8 | ≥ 8 | 2 | 1 | ≤ 1 | ≤ 0.12 | ≤ 0.5 | ≤ 10 |
| SA093 | + | ≥ 0.5 | ≥ 4 | ≤ 0.5 | ≤ 0.5 | 0.25 | ≤ 0.25 | - | ≥ 8 | ≥ 8 | 2 | 1 | ≤ 1 | ≤ 0.12 | ≤ 0.5 | ≤ 10 |
| SA094 | - | ≥ 0.5 | ≤ 0.25 | ≤ 0.5 | ≤ 0.5 | ≤ 0.12 | ≤ 0.25 | - | ≤ 0.25 | ≤ 0.25 | 2 | ≤ 0.5 | ≤ 1 | ≤ 0.12 | ≤ 0.5 | ≤ 10 |
| SA095 | - | ≥ 0.5 | ≤ 0.25 | ≥ 16 | 1 | 1 | ≤ 0.25 | - | ≥ 8 | ≥ 8 | 2 | 1 | ≤ 1 | ≤ 0.12 | ≤ 0.5 | ≥ 320 |
| SA096 | - | ≥ 0.5 | 0.5 | ≤ 0.5 | ≤ 0.5 | 0.25 | ≤ 0.25 | + | ≥ 8 | ≤ 0.25 | 2 | ≤ 0.5 | ≤ 1 | ≤ 0.12 | ≤ 0.5 | ≤ 10 |
| SA097 | - | ≥ 0.5 | ≤ 0.25 | ≥ 16 | 2 | 1 | ≤ 0.25 | - | ≥ 8 | ≥ 8 | 2 | ≤ 0.5 | ≥ 16 | ≤ 0.12 | ≤ 0.5 | ≥ 320 |
| SA098 | - | ≥ 0.5 | ≤ 0.25 | ≥ 16 | ≤ 0.5 | ≤ 0.12 | ≤ 0.25 | - | ≥ 8 | ≥ 8 | 2 | 1 | ≤ 1 | ≤ 0.12 | ≤ 0.5 | 80 |
| SA099 | - | ≥ 0.5 | ≤ 0.25 | ≤ 0.5 | ≤ 0.5 | ≤ 0.12 | ≤ 0.25 | - | ≤ 0.25 | ≤ 0.25 | 2 | ≤ 0.5 | ≤ 1 | ≤ 0.12 | ≤ 0.5 | ≤ 10 |
| SA100 | - | ≤ 0.03 | ≤ 0.25 | ≤ 0.5 | ≤ 0.5 | ≤ 0.12 | ≤ 0.25 | + | ≥ 8 | ≤ 0.25 | 1 | ≤ 0.5 | ≥ 16 | ≤ 0.12 | ≤ 0.5 | ≤ 10 |
| SA101 | - | ≥ 0.5 | 0.5 | ≤ 0.5 | ≤ 0.5 | 0.25 | ≤ 0.25 | + | ≥ 8 | ≤ 0.25 | 2 | ≤ 0.5 | ≥ 16 | ≤ 0.12 | ≤ 0.5 | ≤ 10 |
