# Supplementary material for: Clonality, virulence genes, and antibiotic resistance of Staphylococcus aureus isolated from blood in Shandong, China
Source: BMC Microbiol. 2021 Oct 18;21:281. doi: 10.1186/s12866-021-02344-6 (PMC8522240; doi:10.1186/s12866-021-02344-6)
Supplement: Supplementary file 2 — Additional file 2. [file 12866_2021_2344_MOESM2_ESM.docx]

Table S2 Allelic profiles and molecular characteristics of *S. aureus* isolates collected in this study

| **Strain** | ***arcC*** | ***aroE*** | ***glpF*** | ***gmk*** | ***pta*** | ***tpi*** | ***yqiL*** | **ST** | ***Spa*** | **SCC*mec*** |
| --- | --- | --- | --- | --- | --- | --- | --- | --- | --- | --- |
| SA001 | 1 | 1 | 1 | 1 | 1 | 1 | 1 | ST1 | t127 |  |
| SA002 | 13 | 13 | 1 | 1 | 12 | 11 | 13 | ST15 | t084 |  |
| SA003 | 12 | 3 | 1 | 1 | 4 | 4 | 3 | ST630 | t377 |  |
| SA004 | 19 | 23 | 15 | 2 | 19 | 20 | 15 | ST59 | t1950 |  |
| SA005 | 1 | 1 | 1 | 1 | 1 | 1 | 1 | ST1 | t127 |  |
| SA006 | 3 | 35 | 19 | 2 | 20 | 26 | 39 | ST398 | t571 |  |
| SA007 | 12 | 3 | 1 | 1 | 4 | 4 | 3 | ST630 | t377 |  |
| SA008 | 139 | 2 | 2 | 2 | 6 | 3 | 2 | ST1777 | t318 | III |
| SA009 | 3 | 35 | 19 | 2 | 20 | 26 | 39 | ST398 | t571 | III |
| SA010 | 3 | 35 | 19 | 2 | 20 | 26 | 39 | ST398 | t571 |  |
| SA011 | 19 | 23 | 15 | 2 | 19 | 20 | 15 | ST59 | t163 |  |
| SA012 | 2 | 3 | 1 | 1 | 4 | 4 | 3 | ST239 | t030 | III |
| SA013 | 1 | 4 | 1 | 4 | 12 | 1 | 10 | ST5 | t1084 |  |
| SA014 | 19 | 23 | 15 | 2 | 19 | 20 | 15 | ST59 | t437 |  |
| SA015 | 12 | 4 | 1 | 4 | 12 | 1 | 3 | ST6 | t701 |  |
| SA016 | 12 | 4 | 1 | 4 | 12 | 1 | 3 | ST6 | t701 |  |
| SA017 | 7 | 6 | 1 | 5 | 8 | 8 | 6 | ST22 | t309 |  |
| SA018 | 1 | 4 | 1 | 4 | 12 | 1 | 10 | ST5 | t954 |  |
| SA019 | 19 | 23 | 15 | 2 | 19 | 20 | 15 | ST59 | t441 |  |
| SA020 | 2 | 3 | 1 | 1 | 4 | 4 | 3 | ST239 | t030 | III |
| SA021 | 1 | 4 | 1 | 8 | 4 | 4 | 3 | ST72 | t324 |  |
| SA022 | 1 | 4 | 1 | 4 | 12 | 1 | 10 | ST5 | t002 |  |
| SA023 | 3 | 35 | 19 | 2 | 20 | 26 | 39 | ST398 | t571 |  |
| SA024 | 2 | 3 | 1 | 1 | 4 | 4 | 3 | ST239 | t030 | III |
| SA025 | 3 | 1 | 1 | 8 | 1 | 1 | 1 | ST188 | t189 |  |
| SA026 | 19 | 23 | 15 | 2 | 19 | 20 | 15 | ST59 | t437 | Ⅳa |
| SA027 | 3 | 35 | 19 | 2 | 20 | 26 | 39 | ST398 | t034 |  |
| SA028 | 7 | 6 | 1 | 5 | 8 | 8 | 6 | ST22 | t309 |  |
| SA029 | 13 | 13 | 1 | 1 | 12 | 11 | 13 | ST15 | t084 |  |
| SA030 | 1 | 4 | 1 | 4 | 615 | 1 | 10 | ST5039 | t002 |  |
| SA031 | 19 | 23 | 15 | 2 | 19 | 20 | 15 | ST59 | t163 |  |
| SA032 | 1 | 4 | 1 | 4 | 119 | 1 | 10 | ST965 | t062 |  |
| SA033 | 19 | 23 | 15 | 2 | 19 | 20 | 15 | ST59 | t437 | Ⅳa |
| SA034 | 19 | 23 | 15 | 2 | 19 | 20 | 901 | ST6731 | t437 | Ⅳa |
| SA035 | 3 | 1 | 1 | 8 | 1 | 1 | 1 | ST188 | t189 |  |
| SA036 | 18 | 71 | 6 | 2 | 7 | 58 | 2 | ST707 | t4523 |  |
| SA037 | 3 | 35 | 19 | 2 | 20 | 26 | 39 | ST398 | t571 |  |
| SA038 | 7 | 6 | 1 | 5 | 8 | 8 | 6 | ST22 | t1516 |  |
| SA039 | 1 | 4 | 1 | 4 | 12 | 1 | 10 | ST5 | t5076 | II |
| SA040 | 4 | 1 | 4 | 1 | 5 | 5 | 4 | ST25 | t349 |  |
| SA041 | 3 | 35 | 19 | 2 | 20 | 26 | 39 | ST398 | t571 |  |
| SA042 | 12 | 4 | 1 | 4 | 12 | 1 | 3 | ST6 | t2467 |  |
| SA043 | 4 | 1 | 4 | 1 | 5 | 757 | 4 | ST6732 | t18446 |  |
| SA044 | 19 | 23 | 15 | 2 | 19 | 20 | 15 | ST59 | t437 | Ⅳa |
| SA045 | 3 | 1 | 1 | 8 | 1 | 1 | 1 | ST188 | t189 |  |
| SA046 | 3 | 35 | 19 | 2 | 20 | 26 | 39 | ST398 | t034 |  |
| SA047 | 1 | 4 | 1 | 4 | 12 | 1 | 10 | ST5 | t002 |  |
| SA048 | 6 | 5 | 6 | 312 | 7 | 14 | 5 | ST3969 | t159 |  |
| SA049 | 19 | 23 | 15 | 2 | 19 | 20 | 15 | ST59 | t437 | Ⅳa |
| SA050 | 3 | 35 | 19 | 2 | 20 | 26 | 39 | ST398 | t571 |  |
| SA051 | 7 | 6 | 1 | 5 | 8 | 8 | 6 | ST22 | t5335 |  |
| SA052 | 19 | 23 | 15 | 2 | 19 | 20 | 15 | ST59 | t437 |  |
| SA053 | 1 | 4 | 1 | 4 | 12 | 1 | 10 | ST5 | t2460 | II |
| SA054 | 4 | 1 | 4 | 1 | 5 | 5 | 4 | ST25 | t078 |  |
| SA055 | 19 | 23 | 15 | 2 | 19 | 20 | 15 | ST59 | t437 | Ⅳa |
| SA056 | 7 | 6 | 1 | 5 | 19 | 8 | 6 | ST6663 | t5335 |  |
| SA057 | 19 | 23 | 864 | 2 | 4 | 20 | 6 | ST6762 | t1751 | UA |
| SA058 | 5 | 4 | 444 | 4 | 8 | 6 | 15 | ST6664 | t796 |  |
| SA059 | 7 | 6 | 1 | 5 | 7 | 8 | 3 | ST6665 | t309 |  |
| SA060 | 6 | 5 | 6 | 2 | 12 | 14 | 5 | ST6773 | t2092 |  |
| SA061 | 13 | 13 | 1 | 1 | 7 | 11 | 5 | ST6666 | t346 |  |
| SA062 | 6 | 5 | 6 | 2 | 7 | 14 | 13 | ST6667 | t2091 |  |
| SA063 | 1 | 4 | 1 | 4 | 12 | 1 | 10 | ST5 | t954 |  |
| SA064 | 19 | 23 | 15 | 2 | 19 | 20 | 15 | ST59 | t437 | Ⅳa |
| SA065 | 22 | 1 | 14 | 23 | 12 | 4 | 31 | ST88 | t1764 | UA |
| SA066 | 13 | 13 | 1 | 1 | 12 | 11 | 13 | ST15 | t14014 |  |
| SA067 | 3 | 3 | 1 | 1 | 4 | 4 | 3 | ST8 | t008 | Ⅳa |
| SA068 | 5 | 4 | 1 | 4 | 4 | 6 | 3 | ST7 | t796 |  |
| SA069 | 4 | 1 | 4 | 1 | 5 | 762 | 4 | ST6776 | NT |  |
| SA070 | 12 | 4 | 1 | 4 | 12 | 1 | 3 | ST6 | t701 |  |
| SA071 | 3 | 35 | 19 | 2 | 20 | 26 | 39 | ST398 | t571 |  |
| SA072 | 4 | 1 | 4 | 1 | 5 | 5 | 4 | ST25 | t472 |  |
| SA073 | 13 | 13 | 1 | 1 | 12 | 11 | 13 | ST15 | t084 |  |
| SA074 | 2 | 3 | 1 | 1 | 4 | 4 | 3 | ST239 | t037 | UA |
| SA075 | 1 | 4 | 1 | 4 | 12 | 1 | 10 | ST5 | t668 |  |
| SA076 | 19 | 23 | 864 | 2 | 19 | 20 | 15 | ST6774 | t1751 | UA |
| SA077 | 7 | 6 | 1 | 5 | 8 | 8 | 6 | ST22 | t309 |  |
| SA078 | 790 | 23 | 15 | 2 | 19 | 20 | 15 | ST6733 | t437 |  |
| SA079 | 19 | 23 | 15 | 2 | 19 | 20 | 15 | ST59 | t437 | Ⅳa |
| SA080 | 4 | 1 | 4 | 1 | 5 | 5 | 4 | ST25 | t078 |  |
| SA081 | 3 | 35 | 19 | 2 | 20 | 26 | 39 | ST398 | t571 |  |
| SA082 | 19 | 23 | 862 | 2 | 19 | 20 | 15 | ST6734 | t172 | Ⅳa |
| SA083 | 7 | 6 | 1 | 5 | 8 | 8 | 6 | ST22 | t2336 |  |
| SA084 | 7 | 6 | 1 | 5 | 8 | 8 | 6 | ST22 | t309 |  |
| SA085 | 3 | 35 | 19 | 2 | 20 | 26 | 912 | ST6777 | NT |  |
| SA086 | 3 | 35 | 19 | 2 | 20 | 26 | 39 | ST398 | t7160 |  |
| SA087 | 1 | 4 | 1 | 4 | 12 | 1 | 10 | ST5 | t002 |  |
| SA088 | 794 | 13 | 1 | 1 | 12 | 11 | 13 | ST6763 | t084 |  |
| SA089 | 3 | 1 | 1 | 8 | 1 | 1 | 1 | ST188 | t189 |  |
| SA090 | 7 | 6 | 1 | 5 | 8 | 8 | 6 | ST22 | t7611 |  |
| SA091 | 19 | 23 | 15 | 2 | 19 | 20 | 15 | ST59 | t437 | Ⅳa |
| SA092 | 19 | 23 | 15 | 2 | 19 | 20 | 15 | ST59 | t437 | Ⅳa |
| SA093 | 19 | 23 | 15 | 2 | 19 | 20 | 15 | ST59 | t437 | Ⅳa |
| SA094 | 13 | 13 | 1 | 1 | 12 | 11 | 13 | ST15 | t084 |  |
| SA095 | 1 | 4 | 1 | 4 | 12 | 1 | 10 | ST5 | t13506 |  |
| SA096 | 7 | 6 | 1 | 5 | 8 | 8 | 6 | ST22 | t309 |  |
| SA097 | 1 | 4 | 1 | 4 | 12 | 1 | 10 | ST5 | t002 |  |
| SA098 | 5 | 4 | 1 | 4 | 4 | 6 | 3 | ST7 | t796 |  |
| SA099 | 12 | 4 | 1 | 4 | 12 | 1 | 3 | ST6 | t701 |  |
| SA100 | 3 | 3 | 1 | 1 | 4 | 4 | 3 | ST8 | t024 |  |
| SA101 | 5 | 4 | 1 | 4 | 4 | 6 | 3 | ST7 | t091 |  |
